# Gliadin Peptide P31-43 Induces mTOR/NFkβ Activation and Reduces Autophagy: The Role of *Lactobacillus paracasei* CBA L74 Postbiotc

**DOI:** 10.3390/ijms23073655

**Published:** 2022-03-26

**Authors:** Mariangela Conte, Federica Nigro, Monia Porpora, Claudia Bellomo, Francesca Furone, Andrea Luigi Budelli, Roberto Nigro, Maria Vittoria Barone, Merlin Nanayakkara

**Affiliations:** 1 ELFID (European Laboratory for the Investigation of Food Induced Diseases), Department of Translational Medical Science, Section of Paediatrics, University Federico II, Via S. Pansini 5, 80131 Naples, Italy; maryconte_92@hotmail.it (M.C.); monia.porpora@hotmail.it (M.P.); bellomo.claudia96@gmail.com (C.B.); francescafurone@gmail.com (F.F.); merlinnanayakkara@gmail.com (M.N.); 2 I.T.P. Innovation and Technology Provider s.r.l., Via Bisignano a Chiaia 68, 80121 Naples, Italy; federica.nigro@itpna.it; 3 DICMAPI, University of Naples Federico II, 80125 Naples, Italy; andrealuigi.budelli@unina.it (A.L.B.); rnigro@unina.it (R.N.)

**Keywords:** celiac disease (CD), gliadin peptide P31-43, mTOR/NFkβ activation, *Lactobacillus paracasei* CBA L74 postbiotc

## Abstract

Celiac disease (CD) is an autoimmune disease characterized by an altered immune response stimulated by gliadin peptides that are not digested and cause damage to the intestinal mucosa. The aim of this study was to investigate whether the postbiotic *Lactobacillus paracasei* (LP) could prevent the action of gliadin peptides on mTOR, autophagy, and the inflammatory response. Most of the experiments performed were conducted on intestinal epithelial cells Caco-2 treated with a peptic-tryptic digest of gliadin (PTG) and P31-43. Furthermore, we pretreated the Caco-2 with the postbiotic LP before treatment with the previously described stimuli. In both cases, we evaluated the levels of pmTOR, p70S6k, and p4EBP-1 for the mTOR pathway, pNFkβ, and pERK for inflammation and LC 3 and p62 for autophagy. For autophagy, we also used immunofluorescence analysis. Using intestinal organoids derivate from celiac (CD) patients, we analyzed the effect of gliadin after postbiotic pretreatment with LP on inflammation marker NFkβ. Through these experiments, we showed that gliadin peptides are able to induce the increase of the inflammatory response in a more complex model of intestinal epithelial cells. LP postbiotic was able to induce autophagy in Caco-2 cells and prevent gliadin effects. In conclusion, postbiotic pretreatment with LP could be considered for in vivo clinical trials.

## 1. Introduction

The gut is the largest immune organ in the body [1]. Approximately 100 trillion bacteria are associated with our gastrointestinal tract. The microbiota is considered a “super-organism” and is an integral part of the gastrointestinal tract [2]

Numerous functions are attributed to the microbiota in the human gut; in fact, it competes (for space and nutrients) with potential pathogens, induces the secretion of antimicrobial peptides, and stimulates differentiation and proliferation through interaction with intestinal epithelial cells [3,4], which, in turn, regulate intestinal homeostasis [5,6,7]. In addition, they modulate the inflammatory response induced by nutrient excess as nutrients are modulators of various cellular functions and may be involved in tissue immune response and inflammation.

Celiac disease (CD) is a systemic immune-mediated disease caused by gluten and related prolamins contained in wheat, barley, and rye. It occurs in genetically predisposed individuals and is characterized by a variable combination of gluten-dependent clinical manifestations, the presence of specific autoantibodies in serum, positivity for HLA DQ2, and/or DQ8 haplotypes and enteropathy. To date, the gluten-free diet represents the only therapeutic option for these patients, who must eliminate gluten-containing grains from their diet for life.

Microbial dysbiosis is widely reported in CD patients [8,9]. The activation of innate immunity by gliadin peptides is an important component of the early events of the disease. In particular, the “toxic” A-gliadin peptide P31-43 induces several pleiotropic effects, including epidermal growth factor receptor (EGFR)-dependent actin remodelling and proliferation in cultured cell lines and enterocytes from CD patients and inflammation [10,11].

The mechanistic target of the rapamycin (mTOR) network is an evolutionarily conserved signaling hub that senses and integrates environmental and intracellular nutrient and growth factor signals to coordinate basic cellular and organismal responses such as cell growth, proliferation, apoptosis, and inflammation [12]. Gliadin sustains the mTOR inflammatory response in celiac disease [13]. Moreover, gliadin-derived peptides are able to stimulate enterocytes [14,15] causing the upregulation of proinflammatory cytokine expression such as the activation of the nuclear transcription factor-B (NFkβ) pathway in the small intestinal mucosa of CD patients [16].

Autophagy is crucial for cytoplasmic recycling, fundamental homeostasis, and cell survival [17,18]. It is also an essential component of immune defense against bacterial pathogens such as *Mycobacterium tuberculosis*, *Salmonella enteric*, and *Escherichia coli* [19,20,21]. Thus, triggering autophagy is essential for cell survival during pathogen infection [22,23]. The induction of autophagy involves numerous proteins and multiple signaling pathways. Microtubule-associated light chain 3 protein 1 (LC3, a mammalian homolog of yeast Atg8), vital for autophagosome formation, is considered one of the markers of autophagy [24]. P62 (also known as SQSTM1), is a marker of autophagic flux, is selectively incorporated into autophagosomes, and is efficiently degraded by autophagy [25].

Recent interest was generated by the use of prebiotics, probiotics, and postbiotics to modify the intestinal microbiome. Major interest is focused mainly on the use of postbiotics, a substance released by or produced through the metabolic activity of the microorganism in the gut when feeding on fibrous compounds, which exerts a beneficial effect on the host, either directly or indirectly. The term postbiotic refers to compounds such as protein compounds, hydrogen peroxide (H_2_O_2_), bacteriocins, organic acid, EPS, and enzymes [26,27] These compounds have positive antimicrobial, anticancer, immunomodulation, antioxidant, antihypertensive, cholesterol-lowering, and antiproliferative properties [28,29,30,31,32]. Presently, scientific literature confirms that postbiotic components can be used as promising tools for prevention and treatment strategies in gastrointestinal disorders with less undesirable side effects, particularly in infants and children [33].

In this paper, we analyzed the effect of the gliadin peptide P31-43 on the mTOR and autophagy pathways and the ability of the LP CBA L74 (LP) postbiotic to prevent these effects in Caco-2 cells, an intestinal epithelial cell line, and organoids from intestinal biopsies of CD patients

## 2. Results

### 2.1. Gliadin Peptides Were Able to Induce mTOR, p70S6 Kinase and 4EBP Phosphorylation

The mammalian/mechanistic target of rapamycin (mTOR) is a serine/threonine kinase that integrates a multitude of extracellular signals and intracellular cues to drive growth and proliferation.

**Figure 1:**
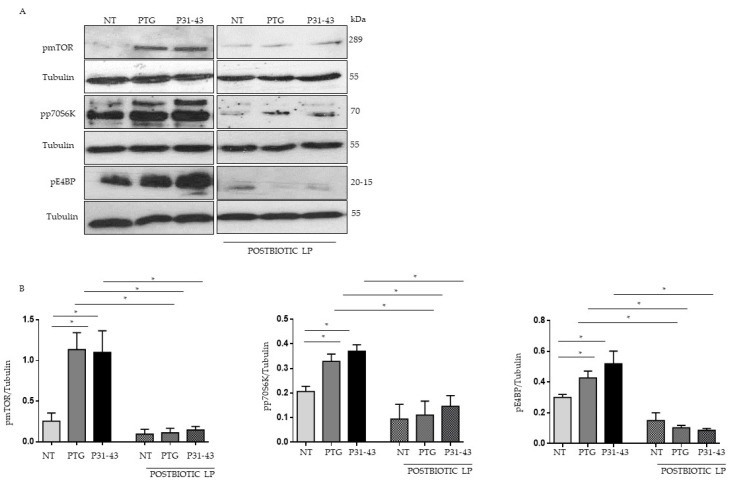
LP postbiotc prevents the gliadin-induced activation of the mTOR pathway in Caco-2 cells. (**A**) Western blot analysis of protein lysates from Caco-2 cells untreated (NT), treated with PTG and P31-43 for 30 min, and pretreated with LP postbiotc for 1 h were blotted with antibodies against pmTOR, pp70S6k, and p4EBP. Tubulin was used as a loading control. The immunoblotting analysis was representative of three independent experiments. (**B**) Densitometric analysis of bands from WB as in A. Columns represent the mean, bars the standard deviation of the relative intensity of pmTOR, pp70S6k, and p4EBP with respect to total tubulin protein. Student’s *t*-test = * *p*  <  0.05.

We investigated the effects of the gliadin peptide P31-43 and PTG (peptic-tryptic digest of gliadin) on the mTOR and autophagy pathway in Caco-2 cells, an intestinal epithelial cell line that is responsive to gliadin. To determine whether the mTOR pathway is active in Caco-2 cells, we analyzed them before and after treatment with PTG and p31-43, the phosphorylated/active form of mTOR and downstream targets p70S6 Kinase 1 (S6K1) and eIF4E binding protein (4EBP), by Western blot.

p-mTOR, p-S6K1, and p-4EBP were increased with respect to untreated cells (NT) after treatment with both p31-43 and PTG. Pretreatment with LP postbiotic was able to prevent the activation of the mTOR pathway in Caco-2 cells (Figure 1).

### 2.2. Gliadin Peptides Reduced the Levels of LC3 and Increased the Levels p62 Autophagy Markers

In the presence of nutrient restriction, mTOR is generally dephosphorylated, and autophagy is induced. The opposite is true in the presence of nutrients. For these reasons, we evaluated the autophagy pathway in Caco-2 cells after treatment with P31-43 and PTG.

**Figure 2:**
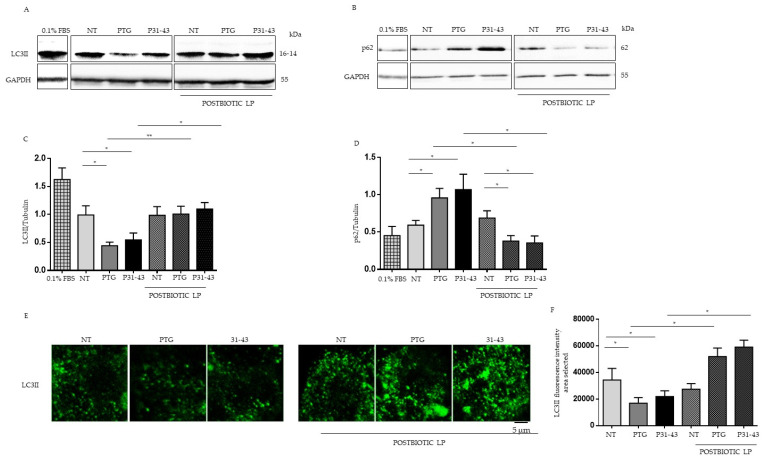
Pretreatment with LP postbiotc increased LC3II expression after treatment with PTG and P31-43. (**A**,**B**) Western blot analysis of protein lysates from Caco-2 cells untreated (NT), treated with PTG and P31-43 for 24 h, and pretreated with LP postbiotc were blotted with antibodies against LC3II and p62. GAPDH was used as a loading control. As a positive control of autophagy induction, the cells were starved with serum at 0.1% FBS. The immunoblotting analysis was representative of three independent experiments. (**C**,**D**) Densitometric analysis of bands from WB as in A. Columns represent the mean, bars the standard deviation of the relative intensity of LC3II and p62 with respect to total tubulin protein. Student’s *t*-test = * *p*  <  0.05, ** *p*  <  0.01. (**E**) Immunofluorescence analysis of anti-LC3II from untreated Caco-2 cells and Caco-2 cells treated with PTG and P31-43 for 24 h and pretreated with LP. Images obtained using a 63× objective (two times digital zoom) were shown. The white bar represents 5 micrometers. (**F**) Statistical analysis of fluorescence intensity of LC3II in the area selected. Student’s *t*-test = * *p*  <  0.05.

Autophagy markers LC3II and p62 protein, a protein necessary for autophagy progression, were evaluated before and after treatment with PTG and P31-43 for 3 (Supplementary Materials Figure S2) and 24 h (Figure 2) by Western blotting in Caco-2 cells. As described by Manai et al. [34], we observed a reduction of LC3II levels and an increase of p62 that was statistically significant only after 24 h of treatment with PTG and P31-43. All this indicates that both the PTG and P31-43 can reduce autophagy and its progression. Interestingly, pretreatment with postbiotic LP prevents the effects of gliadin peptides on LC3 (Figure 2A–C) and p62 levels (Figure 2B–D). Moreover, these data were also confirmed by immunofluorescence (Figure 2E).

In addition, we tested the effect of rapamycin, an inhibitor of mTORC1, on the mTOR/autophagy pathways both in the presence and absence of P31-43 in CaCo-2 cells by Western blotting. Interestingly, rapamycin was able to inhibit the mTOR pathway and increase autophagy (Supplementary Materials Figure S1).

### 2.3. Pretreatment with Postbiotc from LP Decreased NFKβ and ERK Phosphorylation Induced by Treatment with P31-43 and PTG

**Figure 3:**
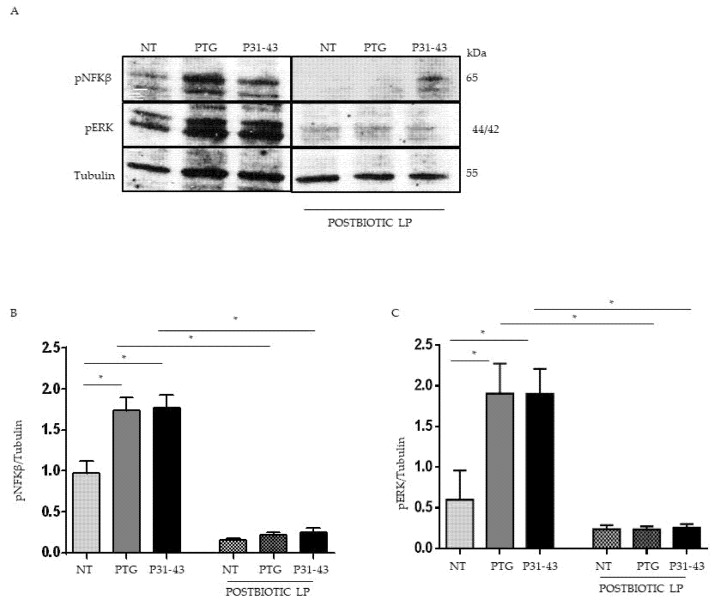
Pretreatment with LP decreased NFkβ and ERK phosphorylation after treatment with P31-43 and PTG. (**A**) Western blot analysis of protein lysates from Caco-2 cells untreated (NT), treated with PTG and P31-43 for 30 min, and pretreated with LP postbiotc for 1 h were blotted with antibodies against pNFkβ and pERK. Tubulin was used as a loading control. The immunoblotting analysis was representative of three independent experiments. (**B**,**C**) Densitometric analysis of bands from WB as in A. Columns represent the mean, bars the standard deviation of the relative intensity of pNFKβ and pERK with respect to total tubulin protein. Student’s *t*-test = * *p*  <  0.05.

Both PTG and P31-43 are able to activate NFkβ [15,35], and PTG is described to activate mTOR [13]. MAPKs are involved in Iκκ-dependent NFkβ activation [36]. We tested the effect of the LP postbiotc on PTG, and P31-43 inflammation markers increased. For this purpose, Caco-2 cells were pretreated with LP postbiotcs for 1 h, and after being stimulated with PTG and P31-43, the activation of NFkβ and ERK was evaluated by WB. Our data showed an increase in NFkβ phosphorylation levels after stimulation with PTG and P31-43. The postbiotcs of LP reduced this phosphorylation (Figure 3A,B). Additionally, ERK activation was increased after PTG and P31-43 treatment [15] and, again, pretreatment with postbiotcs of LP prevented ERK activation (Figure 3A,C).

### 2.4. In CD Intestinal Organoids Lactobacillus paracasei Decreased NFK-β Phosphorylation and Prevented P31-43 Effects

To test the postbiotc LP in a more complex and physiologic cell model, we used intestinal organoids derived from CD patients in the active phase of the disease and control subjects. Organoids were derived from intestinal staminal cells and were cultivated for 4 weeks in 3D before opening them in 2D to allow treatment on the apical part of the epithelial cells.

**Figure 4:**
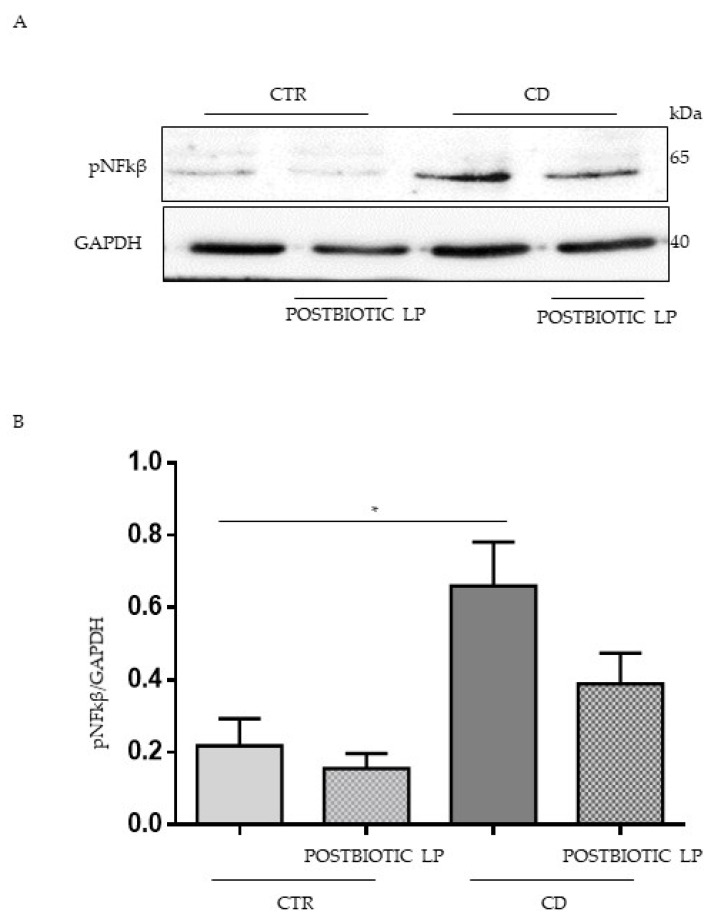
Inflammation in CD organoids can be prevented by LP postbiotic. (**A**) Western blot analysis of protein lysates from organoids from CD patients and controls, treated with LP postbiotc for 3 h were blotted with antibodies against NFkβ. GAPDH was used as a loading control. The number of subjects investigated was three. (**B**) Densitometric analysis of bands from WB. Columns represent the mean, bars the standard deviation of the relative intensity of NFkβ with respect to total tubulin protein. Student’s *t*-test = * *p*  <  0.05.

Intestinal organoids from CD patients were inflamed [37] with respect to the controls, as shown in Figure 4A,B. In these conditions, we tested the effect of the LP postbiotic. Interestingly the LP postbiotc was able to reduce the constitutive inflammation present in CD organoids.

**Figure 5:**
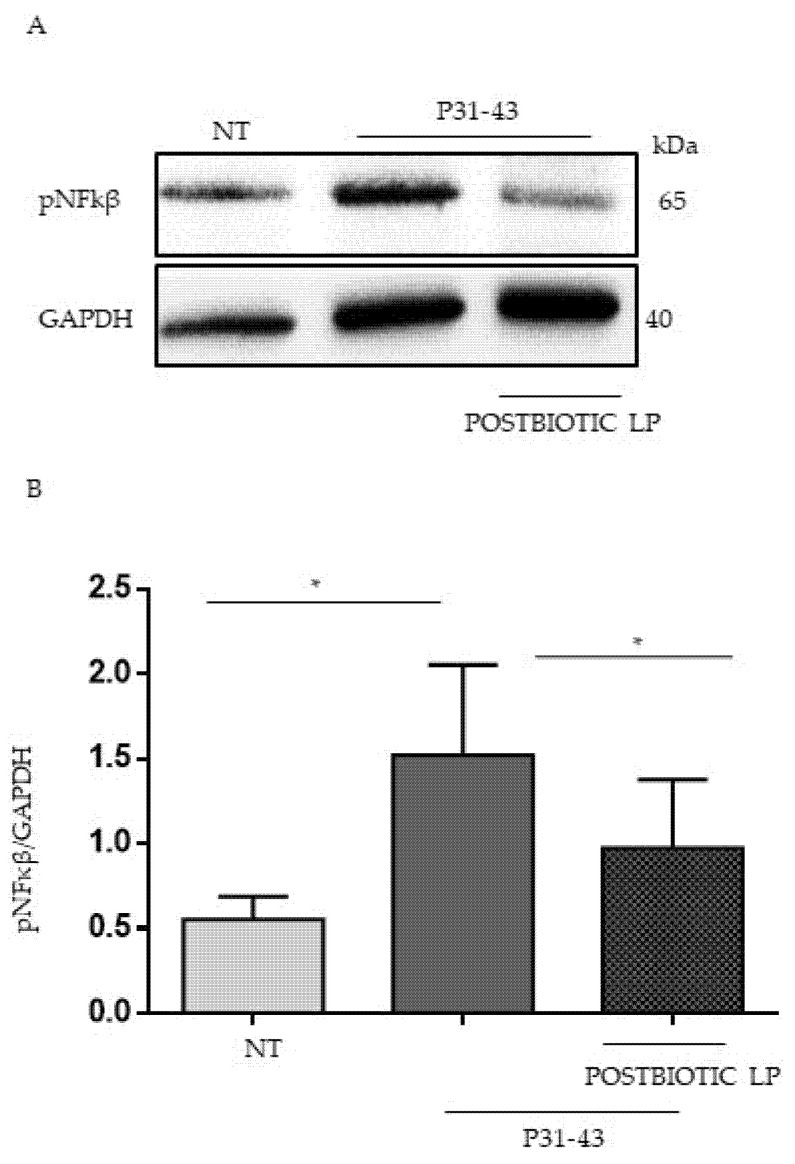
(**A**) Western blot analysis of protein lysates from organoids from CD patients, treated with P31-43 for 1 h and pretreated with LP postbiotc for 2 h, were blotted with antibodies against NFkβ. Tubulin was used as a loading control. The immunoblotting analysis was representative of three independent experiments. The number of subjects investigated is indicated. (**B**) Densitometric analysis of bands from WB. Columns represent the mean, bars the standard deviation of the relative intensity of NFkβ with respect to total tubulin protein. Student’s *t*-test = * *p*  <  0.05.

Next, we tested the effect of P31-43 on CD organoids using as a read out of the inflammation the phosphorylation of NFkβ. As shown in Figure 5A,B P31-43 caused a statistically significant increase in NFkβ phosphorylation; this effect was already described before [15]. LP postbiotc was able to prevent the increase of NFkβ phosphorylation induced by P31-43 on CD organoids.

## 3. Discussion

In this study, we investigated the effects of gliadin on the mTOR/autophagy pathway and inflammation in epithelial cells and intestinal organoids from CD patients. Moreover, we evaluated the ability of the postbiotc *Lactobacillus paracasei* (LP) to prevent these effects.

mTOR is a tyrosine kinase that is able to regulate cell proliferation and inhibit the autophagy pathway. It is regulated by signals, such as growth factors and nutrients, that drive cell growth and proliferation [38]. The autophagy pathway is triggered when mTOR is dephosphorylated/inactivated. It is a pathway closely connected with cell regeneration and consists of an auto-cell digestion process generally activated to remove damaged macromolecules and organelles in order to maintain cellular homeostasis [39]. The activation of mTOR and the consequent deactivation of the autophagy pathway causes the induction of inflammatory markers such as NFkβ [40].

Caco-2 cells, intestinal epithelial cells derived from colon carcinoma and responsive to gliadin, were used to study the effects of gliadin on the mTOR and autophagy pathway. Both the P31-43 peptide and peptic-tryptic digest of gliadin (PTG) were able to activate mTOR and the downstream pathway by activation of the E4BP and 70S6k proteins. Since the activation of mTOR induces a reduction of autophagy [39], we investigated the activity of P31-43 and PTG on autophagy itself. We showed that in the presence of PTG and P31-43, there was a reduction of LC3-positive vesicles and the autophagic flux to the lysosomes was decreased, as demonstrated by the increase in p62 only after 24 h of treatment [34]. Taken together, these data demonstrate that P31-43 and PTG induce a reduction in both autophagic vesicles and their flux to lysosomes. Interestingly, rapamycin was able to inhibit the mTOR pathway and increase autophagy. Furthermore, P31-43 and PTG were able to activate NFkβ in Caco-2 cells

Recent studies investigated the ability of probiotics to prevent the effects of gliadin in vivo. Their effects were studied both in subjects with celiac disease (CD) and in subjects with potential celiac disease [28]. Probiotics seem to have a good effect on CD symptoms when they are present, but they do not seem to have effects on the intestinal lesion.

Taken together, the data on the probiotic effects on CD indicates that the use of probiotics can act on gastrointestinal symptoms, giving an ameliorative effect, but have little effect on the natural history of the disease [28].

More recently, the interest of the scientific world is focused on postbiotcs. A postbiotic is defined as the “preparation of inanimate microorganisms and/or their components: soluble factors secreted by live bacteria or released after their lysis, including various cell surface components, lactic acid, short-chain fatty acids (SCFA), and bioactive peptides that confer a health benefit on the host [41].

At present, no reports are available regarding the use of postbiotics in CD. For this reason, in this study, we investigated the ability of LP postbiotcs to prevent the effects of gliadin and P31-43 peptide on mTOR/autophagy pathways and inflammation in Caco-2 cells. Moreover, we used intestinal organoids from CD patients to test the LP postbiotc effect on inflammation in a more complex and physiologic cell model.

We showed that after pretreatment with the postbiotc LP, both the peptide P31-43 and the PTG effects on mTOR activity and the downstream pathway were prevented. Moreover, pNFkβ was also reduced in the presence of LP postbiotc and PTG or P31-43.

We investigated the activity of P31-43 and PTG on Caco-2 cells pretreated with postbiotc LP on autophagy, demonstrating an increase in autophagy vesicles with the increase in the LC 3 marker and of the autophagic flux to the lysosomes with the reduction of p62.

Although postbiotic LP was able to inhibit the PTG and P31-43 stimulated effects, it is important to note that it also had an effect on the untreated sample. This indicates that the postbiotic LP effect could be independent of, and dominant over the PTG, P31-43 activities. On the other hand, the mechanisms of this activity are not clear, although a possible effect on the intracellular trafficking of the gliadin peptides and also other proinflammatory agents, such as bacteria, cannot be ruled out [9,42].

Recently, Freire R et al., using intestinal organoids developed from duodenal biopsies from both non-celiac (NC) and celiac (CD) patients, analyzed the role of microbiota-derived molecules in modulating the epithelium’s response to gluten. They selected three bacterial bioproducts: butyrate, lactate, and PSA derived from Bacteroides fragilis. All bioproducts exerted a global protective effect by reducing the proinflammatory cytokine secretion triggered by PTG [43]. Using intestinal organoids derived from celiac (CD) patients, we analyzed the effect of P31-43 after pretreatment with LP postbiotc on inflammatory marker NFkβ, demonstrating a reduction. The use in vitro of patient-derived organoids to model CD pathogenesis could be a novel tool to further study CD treatment and prevention.

In conclusion, the LP postbiotc is able to prevent the effects of gliadin peptides on the pathway of mTOR, autophagy, and NFkβ in vitro.

These pre-clinical studies are a good basis for activating clinical trials in celiac patients to prevent the proinflammatory effects of gliadin peptides. In particular, it would be interesting to test the effect of the postbiotc for its ability to prevent disease in potential subjects who have anti-transglutaminase antibodies but have not yet developed the intestinal lesion typical of celiac disease. However, more studies are required to investigate the related safety parameters and biological activity of postbiotics in vivo.

Currently, the only available treatment for a patient with celiac disease is a strict gluten-free diet. Despite patients’ best efforts, some subjects can result in continuous exposure due to the cross-contamination or traces of gluten in food. These risks could, in some cases, compromise the health and quality of life of these subjects. It is, therefore, generally useful to study compounds that can prevent the inflammatory effects of gliadin with the hope of reducing the burden of living with celiac disease and improving long-term health outcomes.

## 4. Materials and Methods

### 4.1. Cell Cultures and Treatments

Human colon adenocarcinoma-derived cells (Caco-2) obtained from Interlab Cell Line Collection (Centro di Biotecnologie Avanzate, Genoa, Italy) were grown in DMEM supplemented with 10% fetal calf serum, 100 units penicillin-streptomycin/mL, and 1 mmol/L glutamine (all these products are Gibco Invitrogen, Milan, Italy). As a positive control of autophagy induction, the cells were starved with serum at 0.1% FBS. Cells were maintained in a humidified atmosphere (95%) of air and 5% CO_2_ at 37 °C. LPS-free synthetic peptide P31-43 (Inbios, >95% purity, MALDI-toff analysis as expected) was obtained by Ultrasart-D20 (Sartorius AG, Goettingen, Germany) filtration. LPS levels were below detection (<0.20 EU/mg), assessed by a commercial QCL-1000 kit (Cambrex Corporation, NJ, USA). P31-43 sequence: LGQQQPFPPQQPY. Peptic-trypticdigests of A gliadin were obtained as described before [44]. The alcohol-soluble protein fraction was extracted from whole flour from wheat (Triticum aestivum, variety Sagittario) and then submitted to digestion as previously described [44]. Prolamin peptic–tryptic (PT) digests indicated as PTG were freeze-dried, lyophilized, and stored at −20 °C until used.

Gliadin peptides were used in the following concentrations: peptic-tryptic digests of gliadin (PTG) 500 μg/mL and P31-43 peptide were used at 100 μg/mL.

### 4.2. Organoids

One to two duodenal biopsies per individual were taken with standard endoscopic EGDS during routine gastroduodenoscopy (Table 1) and placed in an ice-cold 10 mL isolation buffer (5.6 mmol/L Na_2_HPO_4_ (Sigma S7907; Sigma–Aldrich, Milan, Italy ), 8.0 mmol/L KH_2_PO_4_ (Sigma P5655; Sigma–Aldrich), 96.2 mmol/L NaCl (Sigma S5886; Sigma–Aldrich), 1.6 mmol/L KCl (Sigma P5405; Sigma–Aldrich), 43.4 mmol/L sucrose (Fisher BP220-1; Thermo Fisher Scientific, Waltham, MA, USA), and 54.9 mmol/L D-sorbitol (Fisher BP439-500; Thermo Fisher Scientific Milan, Italy) in deionized water. Crypt units were isolated according to the protocol of Yuli Wang et al. [45] with minor variations. Briefly, after 60 min, the biopsy samples were further enzymatically digested with collagenase (2 mg/mL, Sigma–Aldrich, Milan, Italy) in a washing buffer (WB) containing penicillin (100 units mL^−1^), streptomycin (0.1 mg mL^−1^), l-glutamine (2 mM), and FBS (10%, *v*/*v*) in DMEM/F12 with HEPES on ice for 30 min. The digest was filtered through a 70 µm strainer (Falcon, Milan, Italy ), and the strainer was rinsed with an additional 10 mL of WB. Crypts were collected by centrifugation at 500× *g* for 5 min. The supernatant was discarded, the crypts were carefully resuspended in 40 µL of ice-cold Matrigel matrix (Corning 35623, Milan, Italy) to enable three-dimensional growth in 48-well plates; the plates were incubated in a cell culture incubator at 37 °C and 5% carbon dioxide for 10 min to allow the Matrigel to solidify. Afterwards, 300 µL cell culture medium enriched with supplements (CM-S) was added to each well and was replaced every second day. The organoids were used for assays or cryo-preserved at −150 °C. To cryopreserve organoids, they were washed with ice-cold PBS EDTA to remove Matrigel and collected by centrifugation at 500× *g* for 5 min. Organoid pellets were suspended in 1 mL WB, 10% faecal calf serum (FCS, Invitrogen, Milan, Italy), and 10% dimethyl sulfoxide, slowly frozen to −80 °C in a cryo freezing container (Nalgene, Milan, Italy), and then transferred to −150 °C for long-term storage. For further research, the cryopreserved organoids were quickly thawed at 37 °C, transferred to 10 mL WB, centrifuged at 2000× *g* for 5 min, plated with Matrigel, and cultured in CM-S medium. For 2D organoids, organoids were seeded in six wells pretreated with Matrigel diluted 1:40 in phosphate-buffered saline (PBS)

### 4.3. Culture Medium to Maintain Organoids (CM-S)

Mouse l-cells that expressed Wnt3a, R-spondin, and Noggin were commercially purchased (ATCC CRL-3276, Genova, Italy), and a conditioned medium (L-WRN) was prepared according to the instructions and protocol of the manufacturer. Culture medium with supplements (CM-S) was prepared using 50% conditioned L-WRN medium and 50% fresh primary culture media: Advanced DMEM/F-12 (cat.12634-010, Invitrogen, Milan, Italy) 1 mM N-Acetyl-l-cysteine (cat. A7250, Sigma, Milan, Italy), 1 × B-27^®^ supplements (cat.12587-010 Gibco, Milan, Italy), 50 ng/mL Epidermal Growth Factor (cat. PMG8041 Gibco Milan, Italy), 10 mM nicotinamide (cat. N0636, Sigma, Milan, Italy), 10 nM Leu15-gastrin I (cat. G9145 Sigma, Milan, Italy), 500 nM A8301 (inhibitor for ALK4/5/7, cat. 70024-90-7 Sigma, Milan, Italy), 10 µM SB202190 (p38 MAP kinase inhibitor, cat. S7076 Sigma, Milan, Italy), and 10 µM Y-27632 (p160 ROCK inhibitor; cat.1254 Tocris, Milan, Italy) in accordance with the protocols [45,46,47]. The organoids were cultured with a 300 µL culture medium, which was changed every second or third day.

### 4.4. Bacterial Growth Conditions

To prepare the postbiotic, we cultivated L. paracasei-CBA L74 in DMEM supplemented with 10% fetal calf serum and 1 mm glutamine until 10^9^ CFU/mL, as previously described [42,48]. The bacterial culture was then centrifuged at 3000 rpm for 10 min, and the supernatant was filtered through a 0 to 22 micron filter.

### 4.5. LC3 Immunofluorescence Staining

Cells, grown on sterile glass coverslips, were transferred into a 24-well plate and pretreated and not with the postbiotcs for 2 h and then stimulated with PTG and P3-43 for 24 h. After fixation with 3% paraformaldehyde for 5 min at room temperature and permeabilization with 0.2% Triton (Biorad, Milan, Italy) for 5 min at room temperature, Caco-2 cells were stained for 1 h at room temperature with anti-LC3II (Cell signaling Milan Italy) antibody. Secondary antibodies Alexa-488 conjugated (Invitrogen) anti-rabbit for LC3II were added to the coverslips for 45 min at room temperature. The coverslips, after mounting on glass slides, were observed by confocal microscope (LSM 510 Zeiss, Milan, Italy), and images were analyzed with AIS Zeiss software to evaluate the intensity of the fluorescence (FI) of the microscopic field under consideration [49]. The magnification of the micrographs was the same for all the figures shown (63× objective).

### 4.6. Western Blot

After treatments, the Caco-2 cells were washed twice with cold PBS and resuspended in lysis buffer (50 mM Tris-HCl (pH 7.4), 1 mM EDTA, 1 mM EGTA, 5 mM MgCl_2_, 150 mMNaCl, 1% Triton, 1 mM PMSF, 1 mM VO4, 100× Aprotinin, and 50× LAP, all of which were purchased from Sigma, Milan, Italy, except for LAP, which was obtained from Roche, Milan, Italy). The human spheroids were seeded in six multiwells (Corning, Milan, Italy) coated with Matrigel diluted 1:40 in phosphate-buffered saline (PBS) for 3 days. After they were stimulated with P31-43 or PTG and postbiotic were homogenized in tissue homogenization buffer 50 mM Tris–HCl (pH 8), 150 mM NaCl, 5 mM MgCl_2_, 1% Triton, 0.5% sodium deoxycholate, 0.1% SDS, 1 mM PMSF, 1 mM VO4, aprotinin, and LAP. The cell lysates were analyzed using SDS-PAGE with a standard running buffer (25 mM Tris, 192 mM glycine, and 0.1% SDS) and transferred onto nitrocellulose membranes (WhatmanGmbh, Dassel, Germany) using a transfer buffer (25 mM Tris, 192 mM glycine, 0.1% SDS, and 20% methanol, all of which were purchased from Sigma-Aldrich, Milan, Italy). The membranes were blocked with 5% nonfat dry milk and probed with rabbit anti pmTOR, pp70S6k, p4EBP, LC3II (Cell Signaling, Euroclone, Milan, Italy), rabbit anti-pNFkβ (Elabscience, Microtech, Naples, Italy), mouse anti pERK (Santa Cruz, Milan, Italy), and mouse ant-tubulin (Sigma-Aldrich, Milan, Italy). The bands were visualized using ECL (GE Healthcare, Amersham, Buckinghamshire, UK) with exposure times of 2–10 min. The band intensity was evaluated by integrating all the pixels of a band after subtraction of the background to calculate the average of the pixels surrounding the band [10].

### 4.7. Statistical Analysis

Statistical analysis and graphics were obtained from Graph Pad Prism (San Diego, CA, USA). The mean and standard deviation of the experiment was calculated; their significance was evaluated by Student’s *t*-test, only accepting results that showed values of *p*  <  0.05 as significant.

## Figures and Tables

**Table 1:** Patient characteristics.

Patients	Range Age (Years)	Sex	Biopsy (Marsh Classification *)	Serum AntiTG2 (U/mL)	Anti-Endomysial Antibody (EMA)
Controls (N = 3)	10–18	M = 1, F = 2	3 = T0	0–1.5	Negative
GCD-CD (N = 3)	3–15	M = 1, F = 2	1 = T3 c	>50	Positive
			2 = T3 c/b		

* T0: Normal; T1: infiltrative lesion; T3: Flat destructive lesion (b: moderate, c: total).

## Supplementary Materials

The following supporting information can be downloaded at https://www.mdpi.com/article/10.3390/ijms23073655/s1.
