# Supplementary material for: Gliadin Peptide P31-43 Induces mTOR/NFkβ Activation and Reduces Autophagy: The Role of Lactobacillus paracasei CBA L74 Postbiotc
Source: Int J Mol Sci. 2022 Mar 26;23(7):3655. doi: 10.3390/ijms23073655 (PMC8999065; doi:10.3390/ijms23073655)
Supplement: Supplementary file 1 [file ijms-23-03655-s001.zip › ijms-1612773-supplementary.pdf]

Supplemental materials

Gliadin Peptide P31-43 Induces mTOR/NFkβ Activation and Reduces Autophagy: Role of *Lactobacillus paracasei* CBA L74 Postbiotc

Mariangela Conte [1], Federica Nigro [2], Monia Porpora [1], Claudia Bellomo [1], Francesca Furone [1], Andrea Luigi Budelli [3], Roberto Nigro [3], M. Vittoria Barone [1,*] and Merlin Nanayakkara [1]

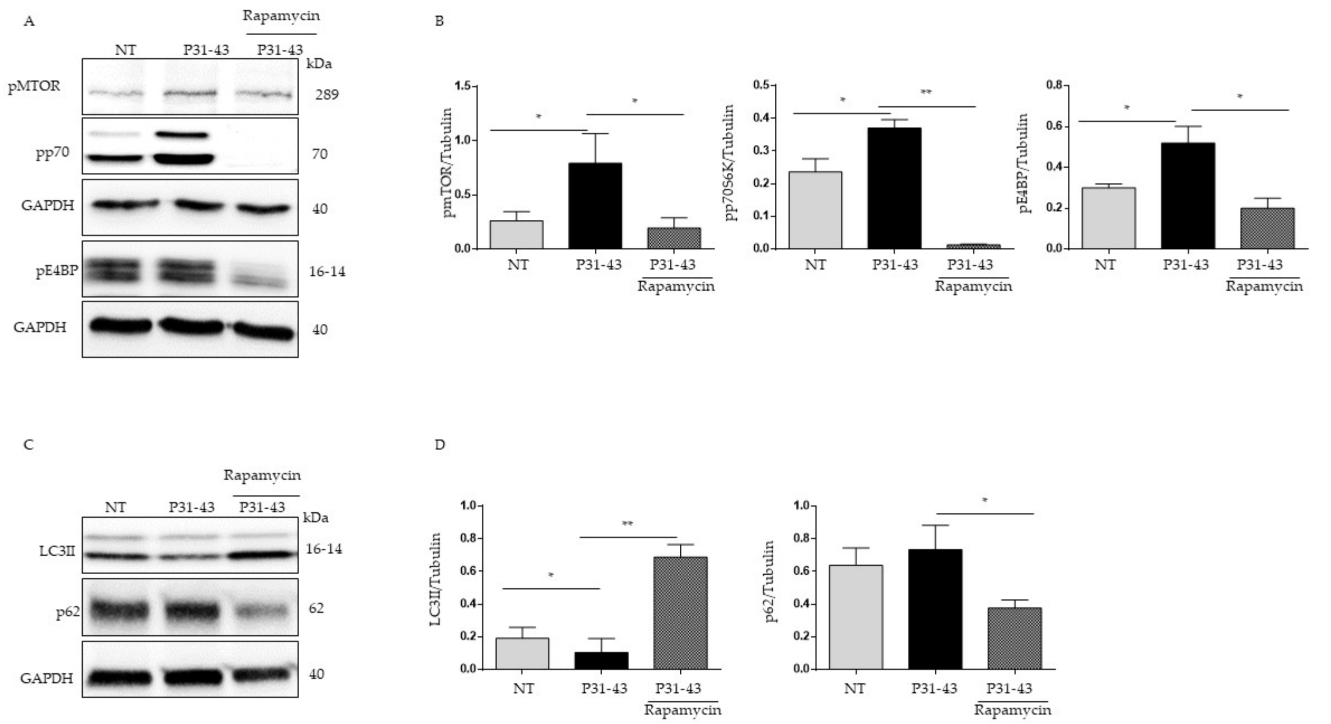

Supplemental Figure S1. Western blot analysis of protein lysates from CaCo-2 cells untreated (NT), treated treated with P31-43 for 24h and pretreated with rapamycin at 100ng/ml concentration for 3h and were blotted with antibodies against (A) pmTOR pp70/pE4BP and (C) LC3 and p62. GAPDH was used as a loading control. The immunoblotting analysis are representative of three independent experiments. (B-D) Densitometric analysis of bands from WB. Student's t-test compared to the untreated (NT) sample, $*p < 0.05$. $**p < 0.01$.

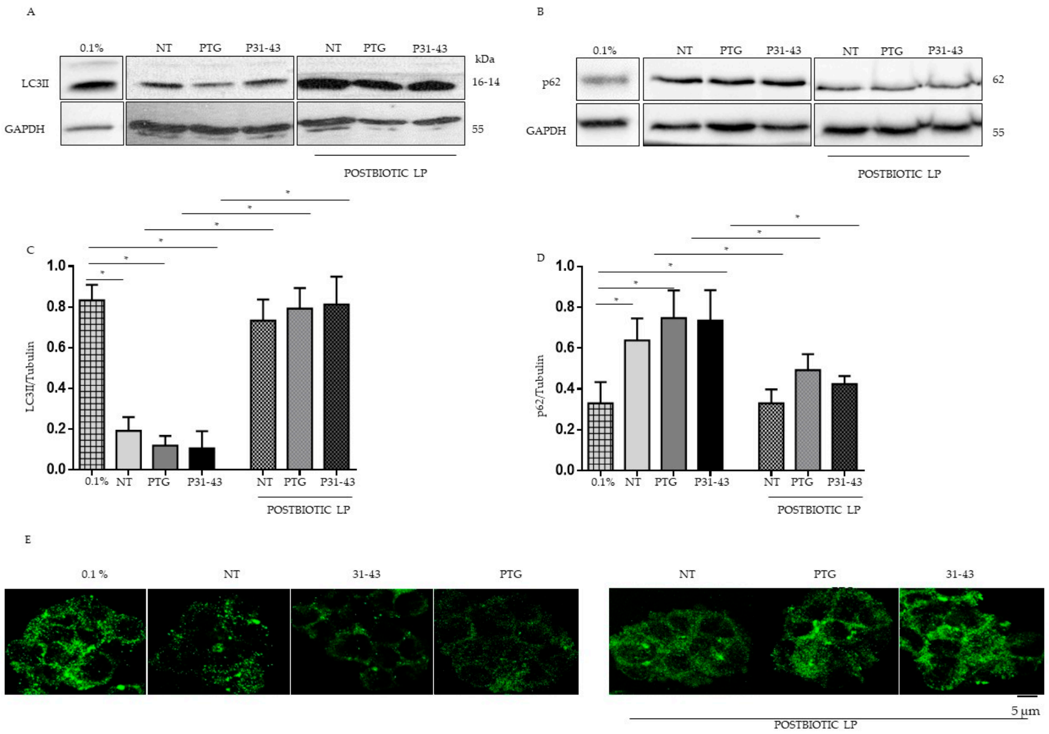

Supplemental Figure S2. Pretreatment with LP postbiotc increased LC3II expression after treatment with PTG and P31-43. As positive control of autophagy induction the cells were starved with serum at 0.1%FBS. (A-B) Western blot analysis of protein lysates from CaCo-2 cells untreated (NT), treated with PTG and P31-43 for 3h and pretreated with LP postbiotc for 2h were blotted with antibodies against LC3II and p62. GAPDH was used as a loading control. The immunoblotting analysis were representative of three independent experiments. (C-D) Densitometric analysis of bands from WB as in A. Columns represent the mean, bars the standard deviation of the relative intensity of LC3II and p62 respect to total tubulin protein. Student's t-test = $*p < 0.05$. (E) Immunofluorescence analysis of anti-LC3II from untreated CaCo-2 cells and CaCo-2 cells treated with PTG and P31-43. Images obtained using a 63 x objective (2 times digital zoom) were shown. White bar represents 5 micrometers
